# Inhibition of ghrelin-induced feeding in rats by pretreatment with a novel dual orexin receptor antagonist

**DOI:** 10.1007/s12576-016-0517-5

**Published:** 2017-01-04

**Authors:** Mariko So, Hirofumi Hashimoto, Reiko Saito, Yukiyo Yamamoto, Yasuhito Motojima, Hiromichi Ueno, Satomi Sonoda, Mitsuhiro Yoshimura, Takashi Maruyama, Koichi Kusuhara, Yoichi Ueta

**Affiliations:** 1 Department of Health and Nutritional Care, Faculty of Medical Science, University of East Asia, Shimonoseki, 751-0807 Japan; 2 Department of Physiology, School of Medicine, University of Occupational and Environmental Health, 1-1 Iseigaoka, Yahatanishi-ku, Kitakyushu, 807-8555 Japan; 3 Department of Pediatrics, School of Medicine, University of Occupational and Environmental Health, Kitakyushu, 807-8555 Japan

**Keywords:** Food intake, Fos, Ghrelin, Immunohistochemistry, Orexin receptor antagonist

## Abstract

Orexin-A and -B, and ghrelin are potent orexigenic peptides. The effects of ACT462206, a novel dual orexin receptor antagonist (DORA), on ghrelin-induced feeding were examined in adult male Wistar rats. Hyperphagia induced by the intracerebroventricular (icv) administration of ghrelin was significantly suppressed for at least 2 h by pretreatment with icv administration of DORA. A marked increase was observed in the number of neurons showing Fos immunoreactivity in the paraventricular nucleus, arcuate nucleus and lateral hypothalamic area (LHA), 90 min after icv administration of ghrelin. Pretreatment with DORA significantly decreased the number of Fos-immunoreactive (IR) neurons; however, Fos immunoreactivity remained significantly increased. Double-immunostaining for Fos and orexin-A showed that many orexin-A-IR neurons in the LHA coexisted with Fos immunoreactivity after icv administration of ghrelin, but their number was reduced significantly by DORA pretreatment. These results suggest that centrally administered ghrelin may activate the orexinergic and non-orexinergic pathways responsible for the regulation of feeding.

## Introduction

Orexin-A and -B discovered as orexigenic neuropetides, were derived from a common precursor, prepro-orexin [1, 2]. They bind to G-protein coupled receptors called orexin receptor type 1 and type 2 (identical to hypocretin receptor-1 and -2), which display 64% homology [1, 2]. Although orexin-A binds to orexin receptor type 1 and type 2 with similar affinities, orexin-B has a 10-fold greater affinity for orexin receptor type 2 than for type 1 [1, 2]. Orexins increase intracellular Ca^2+^ concentration via activation of the Gq/phospholipase C/protein kinase C pathway [1]. Pharmacological blockade of orexin receptor type 1 inhibited feeding and reduced body weight gain in rodents [3]. Genetic mutation in orexin receptor type 2 was found in narcoleptic dogs [4].

Orexin-producing neurons are located mainly in the lateral hypothalamic area (LHA), which is known as the feeding center, and they project their axon terminals to various brain regions, including the hypothalamic nuclei and the brainstem [5–7]. Their receptors are distributed widely in the central nervous system (CNS) [8] and are well-characterized pharmacologically [9]. The deficiency of orexin or orexin receptor causes narcolepsy in mice, dog and humans [4, 10–12]. The linkage revealed between the orexin/orexin receptor system and the regulation of sleep/wakefulness has helped in the development of drugs for sleep disorders. In particular, dual orexin receptor antagonists (DORAs) such as almorexant and suvorexant have been chosen as therapeutic drugs for patients with insomnia [13–15].

Ghrelin, an endogenous ligand for the growth hormone secretagogue receptor (GHSR), was isolated from the stomach [16], and recognized as a potent orexigenic peptide [17–19]. Although ghrelin is predominantly produced and secreted by the stomach, ghrelin-producing neurons and their receptors have been identified in the CNS, including the hypothalamus [17]. Central and peripheral administration of ghrelin increases food intake dramatically in rats and mice [17–20]. Previous studies have demonstrated that central and peripheral administration of ghrelin activates various brain regions, including the feeding-related areas such as the paraventricular nucleus (PVN) and the arcuate nucleus (Arc) in rats [6, 21]. This was observed by Fos immunohistochemistry, which is widely used as an indicator for neuronal activity in the CNS.

Centrally administered ghrelin-induced feeding was significantly attenuated by pretreatment with antibodies against orexin-A and -B [22]. Ghrelin-induced food intake in orexin knockout mice was significantly reduced compared to that in the wild type [22]. These observations suggest that ghrelin-induced feeding may be mediated, at least in part, via the central orexinergic pathway.

Recently, DORAs have been developed for patients with insomnia. To our knowledge, there is a lack of information on the central effects of DORAs on ghrelin-induced feeding. In the present study, we first examined the effects of intracerebroventricular (icv) administration of a novel DORA, ACT462206, which is one of the derivatives from the proline sulfonamide [23], on centrally administered ghrelin-induced feeding in conscious rats. Second, Fos immunohisotochemistry was performed to examine the effects of pretreated ACT462206 on centrally administered ghrelin-activated neurons in the hypothalamus, in particular in the PVN, the Arc, and the LHA in rats. Finally, double-immunostaining for Fos and orexin-A were performed to examine the effects of pretreated ACT462206 on ghrelin-activated orexin-A-producing neurons in the LHA in rats.

## Materials and methods

### Animals

Adult male Wistar rats at 6–8 weeks of age, weighing 250–420 g, were housed individually in plastic cages, in an air-conditioned room (24 ± 1 °C) under a 12-h light (0700-1900)/12-h dark (1900-0700) cycle.

All procedures in the present study were performed in accordance with the guidelines on the use and care of laboratory animals as put forward by the Physiological Society of Japan and under the control of the Ethics Committee of Animal Care and Experimentation, University of Occupational and Environmental Health, Japan.

### Surgical procedures

For icv administration of solutions, all rats were implanted with stainless steel cannulas aimed at the lateral ventricle as previously described [21]. In brief, the rats were anesthetized [sodium pentobarbital, 50 mg/kg body weight, intraperitoneal (ip) injection] and placed in a stereotaxic frame. A stainless steel guide cannula (550-µm outer diameter, 10-mm length) was implanted stereotaxically [24], and the tip of the cannula was 1.0 mm above the left cerebral ventricle. Two stainless steel anchoring screws were fixed to the skull, and the cannula was secured in place by using acrylic dental cement.

After the surgery, the rats were returned to their cages and allowed to recover for at least 5 days. They were handled every day and housed in cages before the start of the experiments.

### Icv administration of solutions

For the icv injection of ACT462206, ghrelin, and saline, a stainless steel injector (300-µm, outer diameter) was introduced through the cannula at a depth of 1.0 mm beyond the end of the guide cannula. The total volume of the solutions of ACT462206 (or saline) and ghrelin (or saline) injected into the lateral ventricle was 5 µL. A novel DORA, ACT462206, [(2*S*)-*N*-(3,5-dimethylphenyl)-1-[(4-methoxyphenyl)sulfonyl]-2-pyrrolidinecarboxamide] was purchased from TOCRIS Bioscience (Bristol, UK). Rat ghrelin was purchased from the Peptide Institute (Minoh, Japan). ACT462206 and ghrelin were dissolved in sterile 0.9% saline.

### Experimental procedures

#### Measurement of food and water intake

To examine the effects of ACT462206 on ghrelin-induced feeding, we administered ghrelin (1 nmol) or saline 30 min after icv administration of ACT462206 (0.1 and 15 nmol) or saline. After icv administration of ACT462206 or saline, the rats were put into metabolic cages at 11:30 am. The dosage of ghrelin was chosen according to our previous study [21]. We measured the cumulative food intake and the cumulative water intake at 0.5, 1, 1.5, 2, 3, 6, and 24 h after icv injection of ghrelin or saline. The number of rats in each group was 4–7.

#### Fos immunohistochemistry and double immunohistochemical staining for Fos and orexin-A

Thirty minutes after the icv administration of DORA (15 nmol) or saline, we administered ghrelin (2 nmol) or saline. Ninety minutes after the icv injection of ghrelin or saline, the rats were deeply anesthetized by ip injection of sodium pentobarbital (50 mg/kg). They were then perfused transcardially with 100 mL of 0.1 M phosphate buffer (PB; pH 7.4) containing heparin (1000 units/L) and 150 mL of a fixative containing 4% paraformaldehyde (PFA). The brains were removed and postfixed with 4% PFA for 48 h at 4 °C. The tissues were then cryoprotected in 20% sucrose in 0.1 M PB for 24 h at 4 °C. For immunostaining, serial section (30 μm thick) were cut using a microtome (REM-700; Yamato Kohki Industrial Co., Ltd., Saitama, Japan). The sections were rinsed twice with 0.1 M phosphate buffered saline (PBS), followed by washing in 0.1 M Tris buffer (pH 7.6) containing 0.3% Triton X-100. Floating sections were incubated with 1% hydrogen peroxide for 60 min, followed by a rabbit polyclonal anti-Fos protein antiserum (#sc-52, Santa Cruz Biotechnology, Santa Cruz, CA, USA) diluted at 1:500 in 0.1 M PBS containing 0.3% Triton X-100 at 4 °C for 4 days. After washing in 0.3% Triton X-100/PBS for 20 min, the sections were further incubated for 120 min with a biotinylated secondary antibody solution (1:250) and finally with an avidin–biotin peroxidase complex (Vector Laboratories Inc., Burlingame, CA, USA) for 120 min. The peroxidase reaction was visualized by incubating the sections in Tris buffer containing 0.02% diaminobenzidine (DAB) and 0.05% hydrogen peroxidase for 3 min. For orexin-A immunostaining, the sections were sequentially incubated with rabbit antisera for orexin-A antibody (lot no. 297980A1, Alpha Diagnostic Intl., Inc., San Antonio, TX, USA) diluted at 1:10,000 in 0.1 M PBS containing 0.3% Triton X-100 at 4 °C for 5 days. The avidin–biotin peroxidase complex was visualized with nickel-sulfate-enhanced DAB. Fos-immunoreactive (IR) neurons were observed as dark brown nuclei, whereas orexin-A-IR neurons were observed as a violet cytoplasmic and axonal precipitate (Fig. 4A). The sections were mounted on gelatin-coated glass slides, and then air-dried, dehydrated in 100% ethanol, cleared with xylene, and covered with a cover slip. The number of Fos-IR and orexin-A-IR neurons of the bilateral hypothalamic area (the PVN, the Arc, and the LHA) was counted in selected six sections according to coordinates given in the rat brain atlas [24] and averaged in all rats. These were counted by two researchers and each group was double-blinded.

### Statistical analysis

Data are represented as the mean deviation from the control (percentage) ± SEM of the cumulative food intake and number of Fos-IR and orexin-A-IR neurons. Each group within an experiment was compared with the saline-treated group. The data were analyzed using a one-way fractional ANOVA followed by Bonferroni and Tukey–Kramer corrections for multiple comparisons. All statistical analyses were performed using JMP 11 (SAS, Tokyo) and SPSS 21 (SPSS, Osaka). The statistical significance was set at *P* less than 0.05.

## Results

### Effects of DORA, ACT462206 on ghrelin-induced feeding

**Fig. 1:**
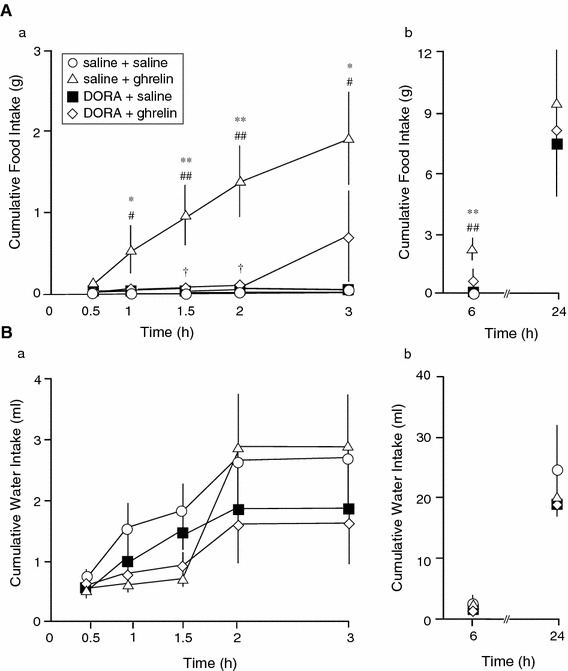
Effects of intracerebroventricular (icv) administration of ghrelin (1 nmol) or saline pretreatment with an orexin receptor antagonist (DORA, ACT462206; 15 nmol) or saline on cumulative food intake in conscious rats (**A**). Effects of icv administration of ghrelin (1 nmol) or saline pretreatment with DORA (15 nmol) or saline on cumulative water intake in conscious rats (**B**). Data for cumulative food intake and water intake are expressed as the mean ± SEM (*n* = 4–7). *P < 0.05 and **P < 0.01, compared with saline + saline-injected rats. ^#^ *P* < 0.05 and ^##^ *P* < 0.01, compared with DORA + saline-injected rats. ^†^ *P* < 0.05, compared with saline + ghrelin-injected rats

Icv administration of DORA at small doses (0.1 nmol) did not suppress ghrelin (1 nmol)-induced feeding (data not shown). Icv administration of DORA (15 nmol) potently suppressed the centrally administered ghrelin-induced feeding at 0.5–2 h after icv administration of ghrelin (1 nmol; Fig. 1Aa). There was no difference among all groups at 24 h after icv administration of the solutions (Fig. 1Ab). Cumulative water intake was comparable among all groups after icv administration of DORA (15 nmol) at all time points (Fig. 1Ba, b).

### Fos-IR neurons in the PVN, the Arc, and the LHA 90 min after icv administration of solutions

**Fig. 2:**
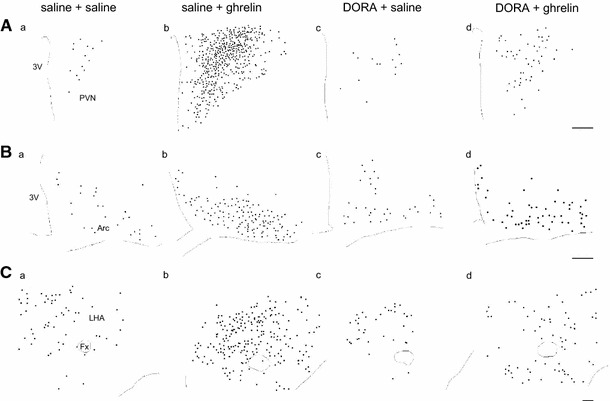
Distribution of Fos-immunoreactive (IR) neurons in the paraventricular nucleus (PVN; **A** *a*–*d*), the arcuate nucleus (Arc; **B** *a*–*d*), and the lateral hypothalamic area (LHA; **C** *a*–*d*) at 90 min after icv administration of ghrelin (2 nmol) or saline, pretreated with orexin receptor antagonist (DORA, ACT462206) or saline in the selected sections. *Circle dots* indicate the localization of the neurons in which Fos-IR was observed. *3V* third ventricle, *Fx* fornix. *Scale bars* indicate 100 μm

The distribution of Fos-IR neurons in the PVN (Fig. 2Aa–d), the Arc (Fig. 2Ba–d), and the LHA (Fig. 2Ca–d) was observed at 90 min after icv administration of the solutions. Icv administration of ghrelin induced Fos immunoreactivity in the entire range of the PVN; in particular, potent increases were observed in the parvocellular division of the PVN (Fig. 2Ab), the ventral part of the Arc (Fig. 2Bb), and the perifornical LHA (Fig. 2Cb). In rats pretreated with DORA before icv administration of ghrelin, the number of Fos-IR neurons decreased dramatically in the PVN (Fig. 2Ad), the Arc (Fig. 2Bd), and the LHA (Fig. 2Cd).

**Fig. 3:**
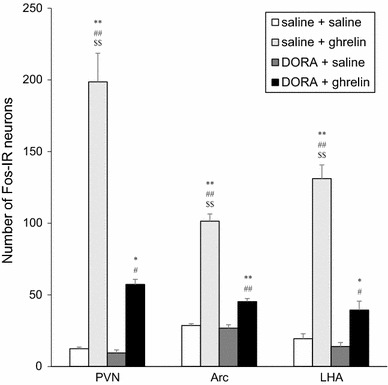
Numbers of Fos-immunoreactive (IR) neurons in the paraventricular nucleus (PVN), the arcuate nucleus (Arc) and the lateral hypothalamic area (LHA) at 90 min after intracerebroventricular (icv) administration of ghrelin (2 nmol) or saline, pretreated with orexin receptor antagonist (DORA, ACT462206) or saline. Data for the number of Fos-IR neurons are expressed as the mean ± SEM (*n* = 4–7). *P < 0.05 and **P < 0.01, compared with saline + saline-injected rats. ^#^ *P* < 0.05 and ^##^ *P* < 0.01, compared with DORA + saline-injected rats. ^$$^ *P* < 0.01, compared with DORA + ghrelin-injected rats

The numbers of Fos-IR neurons in the PVN, the Arc, and the LHA were counted (Fig. 3). Icv administration of ghrelin (2 nmol) caused a significant increase in the number of Fos-IR neurons in the PVN, Arc, and LHA. However, pretreatment of DORA significantly decreased the number of Fos-IR neurons induced by icv administration of ghrelin in the PVN, Arc, and LHA. However, Fos immunoreactivity remained significantly increased in the PVN, the Arc, and the LHA at 90 min after icv administration of ghrelin with DORA when compared to that at 90 min after icv administration of saline without DORA or icv administration of saline with DORA (Fig. 3).

### Effects of DORA, ACT462206 on ghrelin-induced Fos-IR in orexin A-IR neurons

**Fig. 4:**
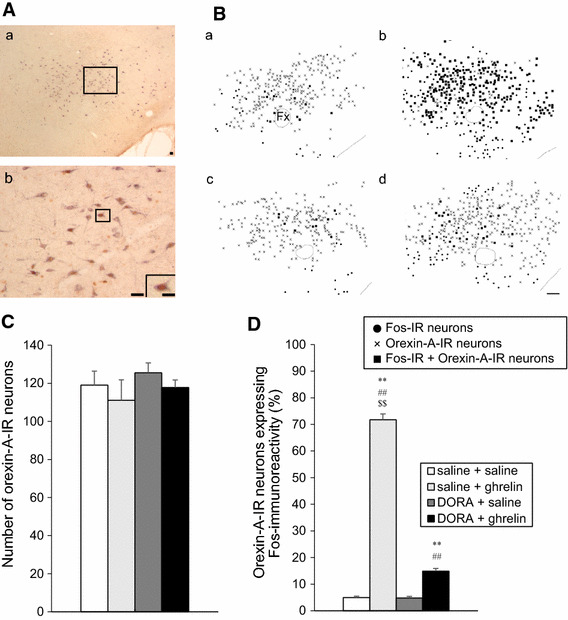
Effect of DORA, ACT462206 (15 nmol) on Fos immunoreactivity in orexin-A- immunoreactive (IR) neurons in the lateral hypothalamic area (LHA) at 90 min after intracerebroventricular (icv) administration of ghrelin (2 nmol). **A** Representative microphotograph showing orexin-A-IR neurons expressing Fos immunoreactivity in the LHA. **A** *b* shows an enlargement of *square* in **A** *a*. The inserted panel in **A** *b* is enlarged from the *square*. *Scale bars* indicate 50 μm (low magnification) and 10 μm (high magnification). **B** Distribution of Fos-IR neurons in the LHA at 90 min after icv administration of ghrelin (2 nmol) or saline, pretreated with DORA (15 nmol) or saline (*a* saline + saline; *b* saline + ghrelin; *c* DORA + saline; *d* DORA + ghrelin). Fos-IR neurons, orexin-A-IR neurons and colocalized neurons are indicated by *circle dots*, *crosses* and *filled squares*, respectively. *Scale bar* indicates 100 μm. **C** Number of orexin-A-IR neurons in the LHA, at 90 min after icv administration of solutions. Data for the number of Fos-IR neurons are expressed as the mean ± SEM (*n* = 5–6). **D** Percentage of orexin-A-IR neurons expressing Fos immunoreactivity in the LHA, at 90 min after icv administration of solutions. Data for the percentage of orexin-A-IR neurons expressing Fos immunoreactivity in the LHA are expressed as the mean ± SEM (*n* = 5–6). *P < 0.05 and **P < 0.01, compared with saline + saline-injected rats. ^#^ *P* < 0.05 and ^##^ *P* < 0.01, compared with DORA + saline-injected rats. ^$$^ *P* < 0.01, compared with DORA + ghrelin-injected rats

The distribution of orexin-A-IR neurons expressing Fos immunoreactivity at 90 min after the icv administration of solutions are shown in Fig. 4A, B.

There was no difference in the number of orexin-A-IR neurons in the LHA at 90 min after the icv administration of solutions (Fig. 4C). The number of orexin-A-IR neurons expressing Fos immunoreactivity significantly increased after icv administration of ghrelin, whereas the number of orexin-A-IR neurons expressing Fos immunoreactivity decreased significantly after icv administration of ghrelin with DORA pretreatment. However, the number of orexin-A-IR neurons showing Fos immunoreactivity remained significantly increased after icv administration of saline without DORA or icv administration of saline with DORA (Fig. 4D).

## Discussion

In the present study, we demonstrated that icv administration of DORA, ACT462206, a novel orexin receptor antagonist, significantly suppressed centrally administered ghrelin-induced feeding in rats. In addition, Fos immunohistochemistry revealed that centrally administered ghrelin induced Fos expression in the PVN, the Arc, and the LHA, which are areas related to feeding behavior. Fos expression was significantly inhibited by pretreatment with DORA; however, it remained significantly increased in those nuclei when compared to that after icv administration of ghrelin without DORA. It is worth noting that Fos expression of orexin-A-IR neurons induced by icv administration of ghrelin was significantly inhibited by pretreatment with DORA, but the Fos expression in orexin-A-IR neurons remained significantly increased.

The suppressive effect of DORA on centrally administered ghrelin-induced feeding suggests that centrally administered ghrelin-induced feeding may be mediated via the central orexin pathway. This result is in agreement with the results of a previous study, which showed that centrally administered ghrelin-induced feeding was significantly attenuated in anti-orexin-A and -B IgG pretreated rats and orexin knockout mice [22].

In the present study, the suppressive effects of pretreatment with DORA (15 nmol) on feeding were observed at 1.5 and 2 h, but not at 3, 6, and 24 h after icv administration of ghrelin. Boss et al. [23] reported that IC_50_ of orexin receptor type 1 and type 2 is 60 and 11 nM, respectively, and t_1/2_ is 1.6–3.1 h after intravenous injection of ACT462206 (1 mg/kg body weight) in rats. These pharmacological characteristics seem to be reasonable to understand the obtained results in the present study.

The possibility that icv administration of DORA decreased the arousal levels, and then inhibited feeding after icv administration of ghrelin in rats has not been excluded. Although we did not investigate the locomotor activity after pretreatment of DORA, the rats pretreated with DORA drank water as same amount as saline pretreated group (Fig. 1Ba, b). Thus, we presume that the pretreatment of DORA did not change locomotor activity, and it may be said that decreased ghrelin-induced food intake with pretreatment of DORA was not due to decreased locomotor activity. Boss et al. also reported that ACT462206 could pass through the blood brain barrier into the CNS [23]. Thus, further studies are required to evaluate whether a peripheral administration, such as intravenous and oral administration, of ACT462206 can inhibit peripherally/centrally administered ghrelin-induced feeding in rats.

GHSR is widely distributed in the CNS, including the hypothalamus [25, 26]. The results of our study as well as previous studies demonstrated that both central and peripheral administration of ghrelin induced Fos expression in various regions, including the hypothalamic nuclei [6, 21]. The hypothalamic nuclei are important sites that regulate feeding and energy metabolism [6, 21, 27–30]. The neurons located in the PVN, the Arc, and the LHA express GHSR [6, 21]. Centrally administered ghrelin may induce Fos expression in those neurons via GHSR directly because the number of neurons expressing Fos immunoreactivity remained significantly increased by pretreatment with DORA (Fig. 3). However, the decrease in number of neurons expressing Fos immunoreactivity in the PVN, the Arc, and the LHA by pretreatment with DORA may be activated by the central orexin pathway via orexin receptors.

Previous studies reported that ghrelin and orexin may have sex differences such as feeding, behavior, and expression levels [31–34]. This evidence suggested that ghrelin-induced feeding via the orexin pathway may have a sex difference. Further studies are required to examine the different effects of ghrelin on orexin-mediated feeding in male and female rats.

In the PVN, corticotropin-releasing hormone (CRH) neurons express orexin receptors [35]. Orexin-containing neurons in the LHA also express CRH type 1 and type 2 receptors [35]. In the present study, pretreatment with DORA attenuated the number of Fos-expressing neurons in the PVN. Although we did not analyze the number of neurons with Fos expression in the parvocellular division of the PVN that contain CRH neurons, Fos-IR neurons induced after icv administration of ghrelin seem to be reduced in the parvocellular division relative to the magnocellular division in the PVN by pretreatment with DORA (Fig. 2Aa, d, Ba, d, Ca, d). The result suggests that orexin receptors may be expressed in CRH neurons in the PVN and is consistent with the previous study [35].

It is hypothesized that centrally administered ghrelin may activate orexin-containing neurons in the LHA via GHSR, and these neurons project their axons to the CRH neurons. CRH neurons in the PVN also project their axons to orexin neurons in the LHA, and activate orexin neurons in the LHA via CRH type 1 and type 2 receptors [35]. This interdependence between orexin neurons in the LHA and CRH neurons in the PVN may be important for stress responses [35].

In the Arc, the neurons synthesize neuropeptides such as neuropeptide Y (NPY)/agouti-related protein (AgRP), which are orexic neuropeptides, and proopiomelanocortin (POMC)/cocaine- and amphetamine-regulated transcript (CART), which are anorexic neuropeptides, and regulate the feeding behavior [25–30]. NPY/AgRP neurons in the Arc express GHSR and orexin receptors [25, 26]. Thus, both orexin and ghrelin activate NPY/AgRP neurons in the Arc, and stimulate feeding. Another possible explanation is that orexin or ghrelin directly activated GABAergic neurons then inhibit POMC/CART neurons in the Arc, which resulted in hyperphagia. The results shown in Fig. 3 are reasonable because pretreatment of DORA significantly attenuated Fos expression in the Arc.

In the LHA, approximately 70% of orexin-A-IR neurons expressed Fos immunoreactivity after icv administration of ghrelin (Fig. 4D). However, pretreatment of DORA significantly reduced Fos immunoreactivity in orexin-A-IR neurons into approximately 15% in the LHA. Neuronal circuits involving the orexin–orexin receptor signaling may exist in the LHA. As orexin-A and -B are derived from a common precursor, prepro-orexin [1, 2], we performed immunohistochemistry only for orexin-A in the present study. However, the detailed mechanism of orexin-A or -B responsible for feeding regulation induced by centrally administered ghrelin remains unclear. We propose investigation of the distinct mechanism of these peptides which are related to feeding regulation, using orexin-A-specific or orexin-B-specific antagonist.

In conclusion, DORA can inhibit centrally administered ghrelin-induced feeding via the central orexin pathway in rats. The physiological relevance of the other pathways activated by centrally administered ghrelin, independent of the orexin pathway, should be clarified by further studies.
